# Elements for optimizing a one-step enzymatic bio-refinery process of shrimp cuticles: Focus on enzymatic proteolysis screening

**DOI:** 10.1016/j.btre.2017.01.003

**Published:** 2017-06-13

**Authors:** R. Baron, M. Socol, R. Kaas, A. Arhaliass, J. Rodriguez del Pino, K. Le Roux, C. Donnay-Moreno, J.P. Bergé

**Affiliations:** a Ifremer, BRM, Rue de l’Ile d’Yeu, BP21105, 44311 Nantes cedex 03, France; b Ifremer, PBA, Rue de l’Ile d’Yeu, BP21105, 44311 Nantes cedex 03, France; c Université de Nantes, GEPEA UMR CNRS 6144, CRTT, Boulevard de l’Université, 44600 Saint-Nazaire cedex, France; d Ynsect, Rue de l’Ile d’Yeu, BP21105, 44311 Nantes cedex 03, France

**Keywords:** Bio-refinery, Shrimp cuticles, Acidic enzymatic proteolysis

## Abstract

This article complements an earlier work published in 2015 Baron et al. (2015) that showed the interest of a shrimp shells bio-refining process. We compare here the effect of eleven commercial proteases at pH 3.5 or 4.0 on a residual amount of shrimp shells proteins after 6 h at 50 °C. The two pH are obtained when respectively 40 and 25 mmol of formic acid are added to 5 g of mild dried shell. Deproteinisation yield above 95% are obtained. Residual amino acids profile in the solid phase was identical for the eleven proteases except for pepsin which was similar to the raw material profile. A significant relative increase in the proportion of Glycine is observed for the ten other cases. Likewise, shapes of size exclusion chromatograms of the dissolved phase are similar except with pepsin.

## Highlights

• Acid Stable Protease (ASP) reached deproteination rates better than 95%, both at pH 3.5 and pH 4.0.
• Five proteases exceed the deproteination rate of 95% at pH 3.5.
• Glycine is less solubilized than the other amino acids except with pepsin enzyme.
• Pepsin and the others enzymes acts differently in two groups in light of the results obtained by exclusion chromatography of aqueous phase.

## Graphical abstract

**Figure:**
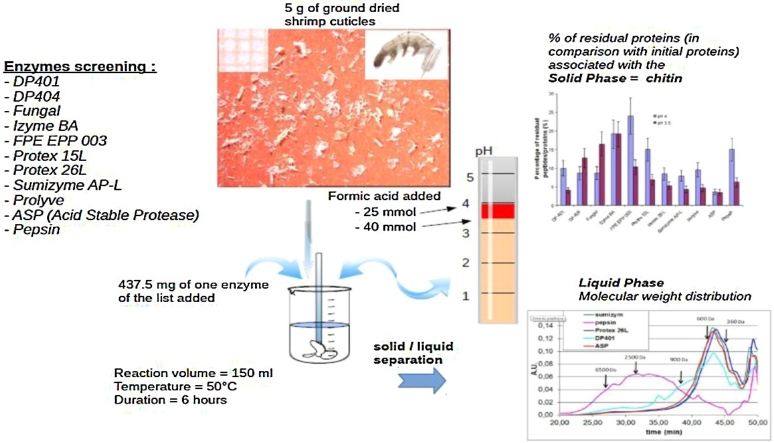

## 1 Introduction

Purification of crustacean chitin shells has been studied by many authors [1], [2], [3], [4], [5], [8], [9], [10], [11], [12], [13], [14], [15], [16], [17], [18], [19], [20], [21], [22] [6], [7] and today represents an important economic activity particularly in the context of shrimp shells value-enhancing schemes [23]. In fact the applications of chitin and its derivatives are more and more widespread. However, the process used is purely chemical and allows only an enhancing value of a small portion of the biomass. Efforts were therefore made to limit the use of chemicals and make this type of purification more sustainable. Bio-refining of crustacean shells, especially shrimp, is an economic, technical and scientific objective already described by some authors [1], [2], [4], [5], [10], [15], [16], [17], [20], [22] [6], [7]. Two biotechnological ways are found in literature: fermentation [5], [10], [16], [17] or enzymatic hydrolysis [1], [2], [4][15], [16], [20], [22] [6], [7]. A bio-refining process in a single step by an exogenous proteolysis in acidic media would enable us to perform chitin purification and deproteination in the same time. Recently, we have shown [1] the promising potential of the bio-refining in a single step of *Litopenaeus vannamei* shrimp shells. The authors have mainly focused on the kinetics of demineralization and the choice of a suitable acid that could ensure a high demineralization yield (>98%) for a pH value close to 4.0 (classical preservation value). Formic acid best fits the selected target criteria. This acid achieves a demineralization yield of 99% at pH 3.5 and 98% at pH 4.0, depending on the selected volume. An increase in solution volume promotes final demineralization. In 6 h, a combination of formic acid and ASP enzyme (Acid Stable Protease), in sufficient concentration, allowed to go beyond the 95% protein removalyield, at pH 3.5 or 4.0. The purity of the obtained chitin is respectively 92% at pH 3.5 and 90% at pH 4.0. The resulting chitin purity over 90%, for a single stage process working in 3.5–4 pH range avoids the additional steps of neutralization of both the solid and dissolved phases.

Here we focus on determining the effectiveness of ten other commercial proteases compared to the ASP enzyme working in 3.5–4.0 pH range. The determination of an enzyme reaching a maximum deproteination yield after 6 h of hydrolysis in 3.5–4.0 pH range, and preferably at pH 4.0 needing less amount of acid, was first sought. The amount of residual proteins was determined using the sum of the quantitative analysis of 16 amino acids. The amino acid profile was also analyzed. The study of size exclusion chromatographs in conjunction with the molecular weight distribution of the generated peptides was conducted on the dissolved phase. All information collected will provide substantial support for the choice of the enzyme.

## 2 Materials and methods

### 2.1 Raw material

The raw material used here corresponds to the *Litopenaeus vannamei* shrimp exoskeleton thawed, peeled by hand, dried, crushed and sieved. The size of the pieces of shell was between 0.5 and 1.0 mm. The protocol for obtaining the raw material is described in the previous article [1].

Composition of the ground cuticle, after mild drying, was: 11.2 ± 2.0% water, 23.4 ± 3.6% minerals (∼1.17 g), 35.0 ± 2.0% proteins (∼1.75 g), 25.2 ± 3.0% chitin (∼1.26 g), and ∼5% others (fatty acids, glycosides, pigments). Composition in brackets are given for 5 g of dried raw material.

### 2.2 Characterization of materials

Ash content was measured gravimetrically, percentages of residual minerals (RM) and demineralization yield (DY) calculated as described in Baron et al. [1]. Protein content is obtained by summing the concentrations of 16 amino acids which were identified, percentages of residual proteins (RP) and deproteination yield (PY) were calculated according to Baron et al. [1].

### 2.3 Experimental setup and samples preparation

For experiments, a fixed initial weight of 5.0 g of mild dried shrimp cuticles was used in a preset volume of acid solution (150 mL) under constant continuous stirring (300 rpm) with magnetic stirrers. Temperature was controlled at 50 °C with thermostatic plates.

Each time point corresponded to a specific test with 5.0 g of cuticle and the whole reaction volume (solid and liquid phases) was collected to ensure the consistency and accuracy of the results. All the solids were removed by filtering with Nylon filters of mesh size 300 μm. Reaction on solids was stopped by rinsing abundantly with 500 mL of distilled water.

Formic acid was purchased from Sigma-Aldrich (Saint-Quentin Fallavier, France).

The molar ratio needed to obtain a desired pH value at 50 °C is estimated, in a first approximation, by Henderson’s relation (calcium carbonate representing more than 90% of minerals [1]).

$$K_{a}=\frac{[{HCOO}^{-}]\;[H_{3}O^{+}]}{\left[HCOOH\right]}\;and\;pH={pK}_{a}+\log_{10}\left(\frac{\left[{HCOO}^{-}\right]}{\left[HCOOH\right]}\right)\;with\;pKa=3,75\;at\;2{}^{\circ}C$$

Solution pH was measured with an analytical pHmeter (CyberScan pH/Ion 510, Eutech Instruments) and with an electrolytic pH electrode (InLab pro expert, Mettler Toledo).

### 2.4 Enzymes

Enzyme activities are either not identical, or expressed in different units, or not supplied by the manufacturer. This makes it difficult to determine the amount of enzyme to be added in order to carry out this comparative work. We have chosen to work with a sufficient amount of enzyme with a weight to weight ratio of enzyme/proteins of 25%.

For 5 g of shell, 1.75 g of proteins is assumed to be present (see Section “raw material”). 437.5 mg of enzyme (=25% of 1.75 g) are added 5 min after shells were poured in 150 mL reaction volume.

### 2.5 Weight distribution analysis of peptides generated after hydrolysis

Twenty milligrams of lyophilized aqueous phase samples from the hydrolysates were eluted in 10 mL solvent: 30% acetonitrile/0.1% trifluoroacetic acid, and were then centrifuged at 10,000*g* during 10 min in a Beckman Coulter Avanti J-25 refrigerated at 10 °C. The sludge and the soluble fraction were then separated [24].

Peptides molecular weight distributions of the soluble fraction were determined by gel filtration chromatography on a FPLC Superdex Peptide 10/30 GL column (Pharmacia Biotech): exclusion size range of 100 − 7.000 Da, eluting solvent (previously defined). The flow rate was 0.5 mL/min. Detection signal was performed with a Diode Array Detector DAD Shimadzu SPD M20A. Detection of peptide bonds was preferentially measured at an absorbance of 205 nm. Standards injected were Glycine: Gly (72 Da), Gly–Gly (132 Da), Gly-Gly–Gly (189 Da), Gly-Gly-Gly-Gly–Gly (303 Da), Leupeptin (463 Da), Substance P (900 Da), Neurotensin (1673 Da), Insulin Chain B (3496 Da), Aprotinin (6511 Da).

A calibration curve between retention time and peptide weight was established using standard peptides in triplicates.

## 3 Results and discussions

The relation between molar ratio and experimental pH value after 6 h at 20 °C was sketched in [1]. On this basis and using the tendency given by the Henderson equation, we approximated the relationship at 50° C by first examining the pH obtained after a 6 h reaction time, for quantities of formic acid, respectively, 25, 30, 35 and 40 millimoles, added to 150 mL of water (i.e. the respective molar ratios ranging from 1.12 to 1.79). A linear relationship of the form *pH* = −0.74*MR+4.83 fairly approximates pH as a function of MR (R^2^_adj_ = 0.98). The Molar ratio needed to obtain the desired pH (around 3.5 or 4) is respectively MR = 1.78 and MR = 1.12.

An important increase of pH is observed in the first 15 min and, after this period, the pH increases very slowly (around 0.01 pH unit per hour). Indeed, for a molar ratio MR = 1.78 at 50 °C, the pH values after 15 min, 1 h, 2 h and 6 h were respectively 3.46, 3.48, 3.49 and 3.53. This result is very advantageous for the one-step enzymatic proteolysis process because pH remains constant during reaction time (close to the assumed optimal pH for enzymatic activity and preservation of dried aqueous phase).

### 3.1 Enzymatic screening at pH 4.0 and at pH 3.5

**Fig. 1:**
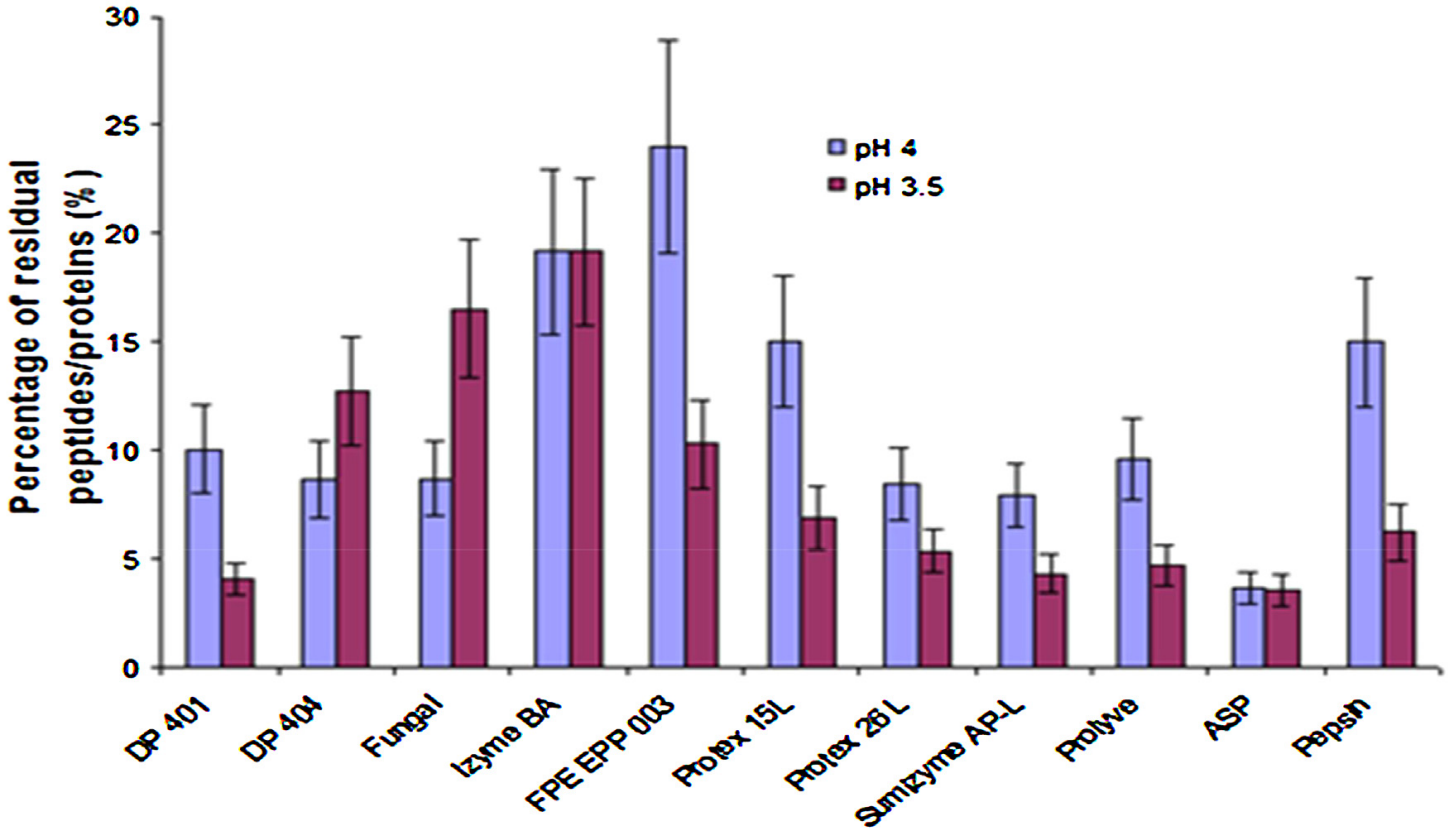
Completeness of enzymatic digestion of proteins (as% of solid phase residual peptides (RP)) after 6 h acidic enzymatic hydrolysis of 5.0 g of shell. 11 enzymes were tested at pH 3.5, 4.0 at 50 °C. 5.0 g of shell containing initially approximately 1.75 g proteins were used in each experiment (Volume = 150 mL, enzyme added = 437.5 mg).

In order to compare deproteination yields, eleven enzymes (see Table 1) were tested in formic acid media at pH 4.0 (molar ratio MR = 1.12) and pH = 3.5 (MR = 1.78) and at a temperature of 50 °C (a majority of enzymes presented a high activity at this temperature – see Table 1) in a predefined volume solution (150 mL). Results are shown in Fig. 1.

**Table 1:** Enzyme characteristics and properties.

Enzyme	Micro-organism or other	Type of enzyme	Society	Optimal pH^a^ (pH range)	Optimal T^a^ (T range)
DP 401 2100 SAPU/g	–	Acid fungal protease	Valley Research (DSM)	3 (2–4)	45–50
DP 404 542000 HUT/g	–	Acid fungal protease	Valley Research (DSM)	4 (2,5–6,5)	50–55
Fungal Protease 500000 HU/g	Aspergillus oryzae	–	Bio-cat	3 (3–6)	50 (25–60)
Izyme BA 0.15 AU/g	–	Aspartate protease	Novozyme	3 (2–4)	50
FPE EPP 003	–	–	DSM	(3–5)	35
–					
Protex 15 L 1000 SAPU/g	Trichoderma reesei	–	Genencor	(4–5)	
Protex 26 L 2000 SAPU/g	Aspergillus niger	–	Genencor	(3,5–4,5)	
Sumizyme AP-L 2000 U/g	Aspergillus niger	Endopeptidase	Shin Nihon Chemical Co	3 (3–5)	60 (50–60)
Prolyve Pac	Aspergillus niger	–	Lyven	3 (2–4)	60 (50–65)
–					
ASP 3000 SAPU/g	Aspergillus niger	–	Bio-cat	2,5 (2–3,5)	30–60
Pepsin 56000 U/g	Gastric mucosa	Peptidase	Sigma-Aldrich	2 (2–4)	37 (30–50)

- not defined.

a for a specific substrate mentioned in their technical document.

Two preliminary assays without enzymes were realized at pH 3.5, 4.0 and 7.0, temperature was 50 °C. Residual protein percentages were 75.0 ± 5.0%, 77.1 ± 5.2% and 77.9 ± 5.2% respectively meaning there is no significant difference in residual proteins in solid-phase whatever the pH tested. But the amount of protein extracted when adding enzymes is significantly higher for all assessed proteases as well at pH 3.5 then 4.0.

At pH = 4.0, the average residual minerals percentage was 2.0 ±0.3% and only one enzyme (ASP) lead to less than 5% residual peptides/proteins. Seven enzymes (DP401, DP404, Fungal, Protex 26L, Sumizyme, Prolyve, ASP) rendered deproteination yields superior to 90%.

At pH = 3.5, the final percentage of minerals was 0.48 ± 0.1% and residual aminoacids percentages were around 5% for five enzymes (DP401, Protex 26L, Sumizyme, Prolyve, ASP).

The amount of peptide/protein recovered in liquid phase is only slightly lower at pH 4.0 than at pH 3.5 with a relative difference of only 4.3%. The amount of minerals that pass in liquid phase is almost complete with a slightly lower value at pH 4.0 with a relative difference of 1.6%. Meanwhile, the amount of acid consumed is 60% lower at pH 4.0.

For both pHs, even though important differences between residual amino acids percentage were found to be from 4 to 24%, this didn’t increase the percentage of residual minerals dispersion (±0.3%), meaning that the degree of demineralization (DY) is not linked to the degree of deproteination (PY).

At pH 4.0 with ASP enzyme, 1.23 ± 0.14 g of chitin containing 0.08 g proteins and 0.02 g of minerals, forming the residual solid, was obtained after filtration. These values indicate, for a biotechnological process, a high degree of purification (92.5%) of the chitin [4]. Moreover, during the process involving chitin transformation into chitosan, its deacetylated derivative, the residual peptides are easily eliminated [5] which allows achieving purity levels of over 98%.

### 3.2 Amino acids distribution in residual solid

**Fig. 2:**
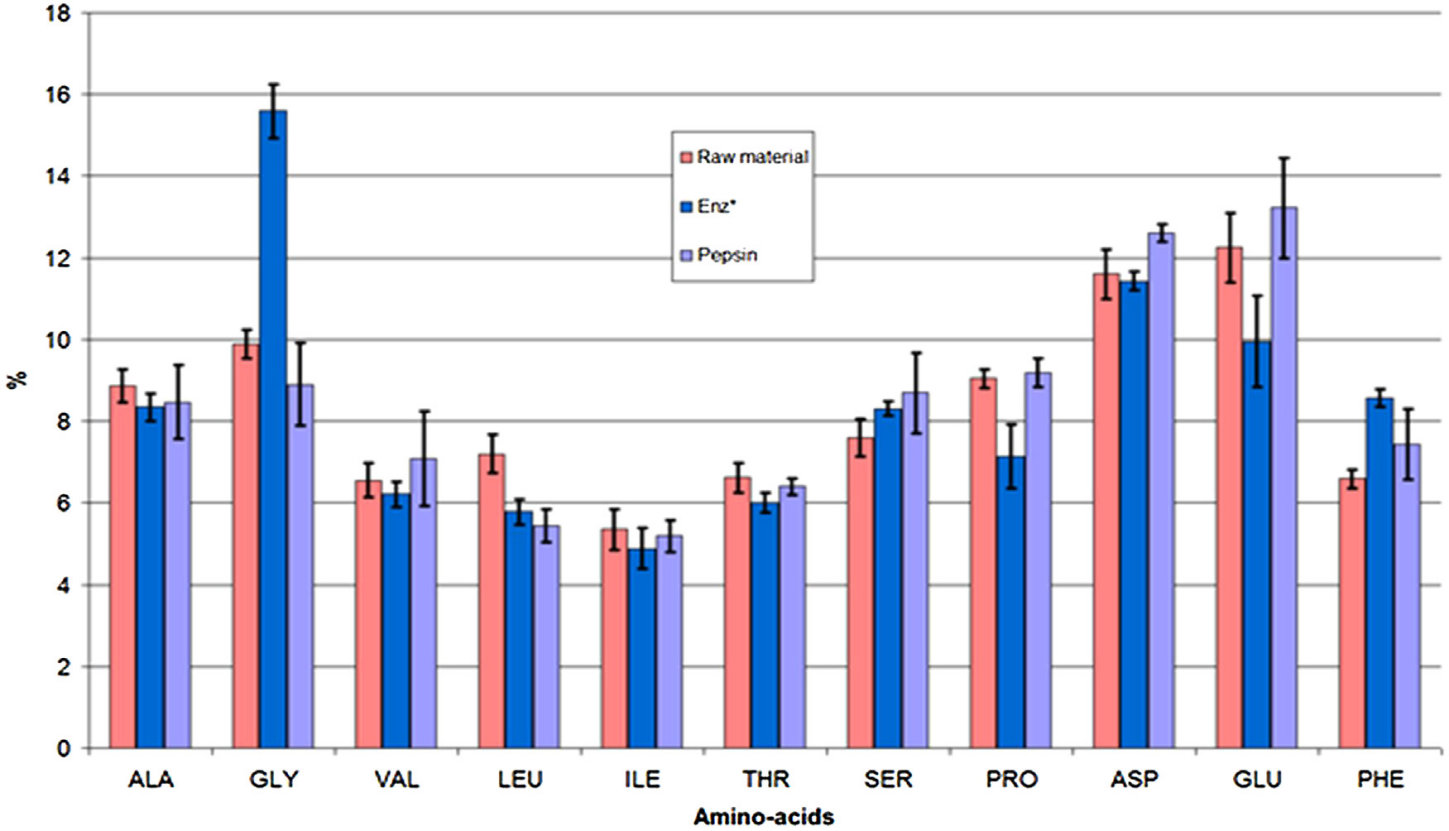
Relative amino-acids composition found in the solid phase (total being the sum of the 16 analyzed amino-acids) after 6 h of enzymatic reaction (pH 3.5 and pH 4.0 at temperature of 50 °C). The average values obtained for the 10 enzymes (all experimented enzymes of Table 1 except pepsin), together with their confidence intervals (CI), are presented in blue. The pepsin hydrolysis results are marked in sky blue and for raw material, amino-acids relative representation is shown in red. Only 11 major amino-acids are shown (amino-acids with a level below 3% were discarded).

In order to compare amino-acids composition of hydrolysates (nutraceutic value) obtained using 10 commercial enzymes and pepsin at pH 3.5, we analyzed the amino-acids composition of the residual solid once the enzymatic reaction had taken place. Results are shown in Fig. 2.

Amino-acid profiles obtained at 50 °C are very similar for all enzymes originating from micro-organisms. A significant difference, when compared with pepsin result, was observed for glycine percentage (higher than 5%). Amino-acid composition, obtained at pH = 3.5 and 50 °C using pepsin, was similar to the raw material.

For all enzymes used in this study, working at pH 4.0 did not significantly affect the observed amino acids composition compared to pH 3.5.

All human essential amino-acids are present in shrimp shell in important proportions when compared with those existing in human proteins, except for methionine which represented around 0.7% relative to total quantity of amino-acids. The total of human essential amino-acids represented about 39% of all amino-acids analyzed in shrimp shell, meaning approximately 0.7 g in 5 g of raw material. This percentage is very close to that of the soybeans (39,31%) which contain between 40 and 45 percent of proteins and have a nutritional quality higher than wheat if we consider their chemical score (FAO, technology of production of edible flours and protein from soybean).

### 3.3 Analysis of molecular weights distribution of peptides from solution by exclusion size chromatography

In order to smooth the effect of the amount of extracted peptides and signal intensity fluctuations observed during repetitions of experiments, the signal was normalized by calculation on the basis of the area under the curve between the retention times from 20 to 50 min.

**Fig. 3:**
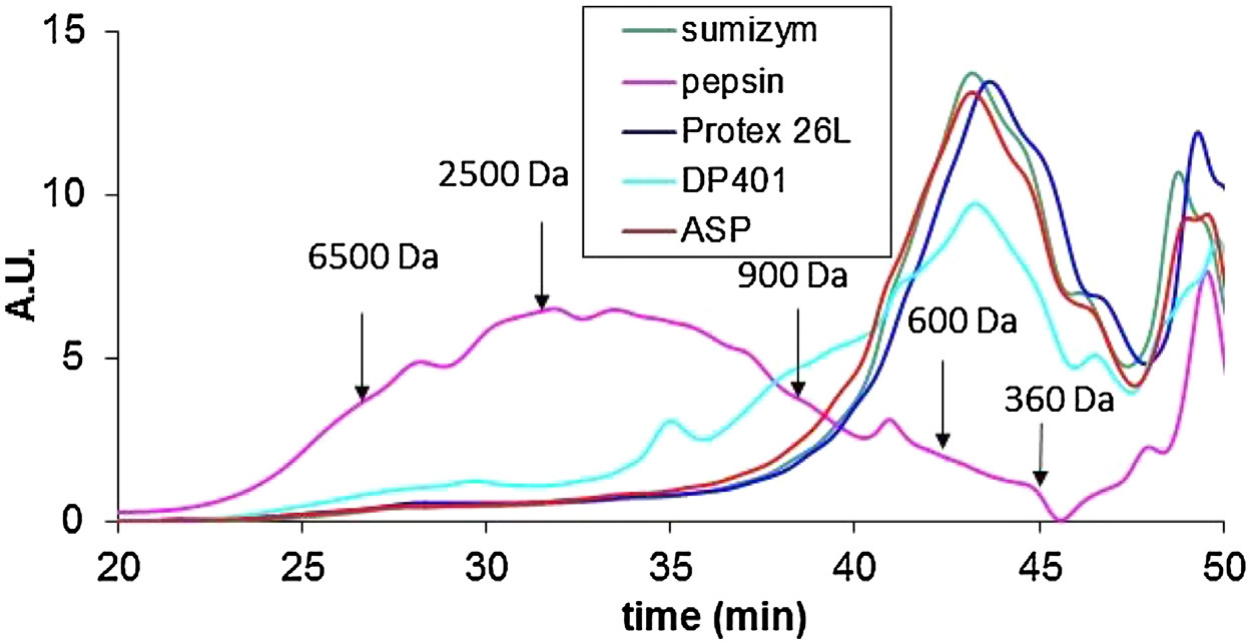
Normalized absorbance intensity at 205 nm versus retention time in exclusion size chromatography for five enzymes (after 6 h of hydrolysis at pH 3.5 and temperature of 50 °C).

Fig. 3 illustrates the two major categories of molecular profiles observed.

The profile of DP401 was chosen to illustrate the maximum dispersion observed with 10 enzymes besides pepsin. The average profile for the class of “fungal” enzymes is shown by the proximity of curves obtained for the Sumizym, protex 26L enzymes and Asp. The molecular profile when using ASP, 26L protex or Sumizym presents only a very small proportion of peptides below 900 Da. Peptides showed mainly sizes between 400 and 600 Da. The profile obtained with pepsin is clearly different. Its distribution is more spread out and starts at much shorter retention times. This curve is characterized by a maximum size of peptides of around 2000 Da. A significant proportion of peptides is larger than 6500 Da. Conversely, the proportion of peptides around 360 Da is very low.

This profile is similar to those observed in previous authors work for lower pH at 40° C with pepsin and formic acid (unpublished data) and retranscribed in size class in [2]. Those previous results demonstrate that increasing hydrolysis time to 12 h or 24 h does not alter the molecular profile and does not significantly reduce the amount of residual proteins. The profile we observe is therefore comparable to that obtained in steady state.

It is thus clear that for our matrix, enzymes cleavage sites are different in the case of pepsin compared to the other enzymes tested. The use of pepsin alone does not allow to obtain a significant proportion of small peptides (from 2 to 4–5 amino acids), unlike the other enzymes tested.

Considering that on one hand biological activity, especially antimicrobial activity, is increased for peptides weighting between 2000 and 300 Da [25], [26], [27], and that, on the other hand, digestibility of the hydrolyzate is facilitated by small sizes [28], it is clearly preferable to use “fungal” enzymes instead of pepsin alone.

## 4 Conclusion

With regard to protein extraction yields, the degree of purification of chitin ( > 92%), the amount of acid used (25 mmol) and the specifications generally required to utilize the soluble fraction of the hydrolyzate in animal feed (digestibility, nutritional value, mineral content), the results obtained with the “fungal” ASP enzyme at pH 4.0 are the most favorable outcome for the implementation of the bio-refinery process in one step proposed by the authors.
